# Effect of Protonation State and *N*-Acetylation of Chitosan on Its Interaction with Xanthan Gum: A Molecular Dynamics Simulation Study

**DOI:** 10.3390/md15100298

**Published:** 2017-09-25

**Authors:** Suha M. Dadou, Musa I. El-Barghouthi, Samer K. Alabdallah, Adnan A. Badwan, Milan D. Antonijevic, Babur Z. Chowdhry

**Affiliations:** 1 Department of Pharmaceutical, Chemical and Environmental Science, Faculty of Engineering and Science, University of Greenwich, Medway Campus, Chatham Maritime, Kent ME4 4TB, UK; S.M.Dadou@greenwich.ac.uk (S.M.D.); M.Antonijevic@greenwich.ac.uk (M.D.A.); B.Z.Chowdhry@greenwich.ac.uk (B.Z.C.); 2 Department of Chemistry, The Hashemite University, P.O. Box 150459, Zarqa 13115, Jordan; musab@hu.edu.jo (M.I.E.-B.); samer.olimat@yahoo.com (S.K.A.); 3 The Jordanian Pharmaceutical Manufacturing Company (PLC), Research and Innovation Centre, P.O. Box 94, Naor 11710, Jordan

**Keywords:** chitosan, xanthan gum, reducing/non-reducing residues, electrostatic interactions, docking, molecular dynamics simulation

## Abstract

Hydrophilic matrices composed of chitosan (CS) and xanthan gum (XG) complexes are of pharmaceutical interest in relation to drug delivery due to their ability to control the release of active ingredients. Molecular dynamics simulations (MDs) have been performed in order to obtain information pertaining to the effect of the state of protonation and degree of *N*-acetylation (DA) on the molecular conformation of chitosan and its ability to interact with xanthan gum in aqueous solutions. The conformational flexibility of CS was found to be highly dependent on its state of protonation. Upon complexation with XG, a substantial restriction in free rotation around the glycosidic bond was noticed in protonated CS dimers regardless of their DA, whereas deprotonated molecules preserved their free mobility. Calculated values for the free energy of binding between CS and XG revealed the dominant contribution of electrostatic forces on the formation of complexes and that the most stable complexes were formed when CS was at least half-protonated and the DA was ≤50%. The results obtained provide an insight into the main factors governing the interaction between CS and XG, such that they can be manipulated accordingly to produce complexes with the desired controlled-release effect.

## 1. Introduction

Over the past three decades, hydrophilic polysaccharides have gained wide scientific interest in relation to drug delivery systems. This is due, in part, to the fact that they are naturally occurring polymers of low toxicity, exhibit high stability, are biocompatible/biodegradable, and show mucoadhesion properties [1,2]. In addition, the structure of polysaccharides can be relatively easily modified in order to synthesize derivatives with desirable characteristics for drug delivery [3].

**Figure 1:**
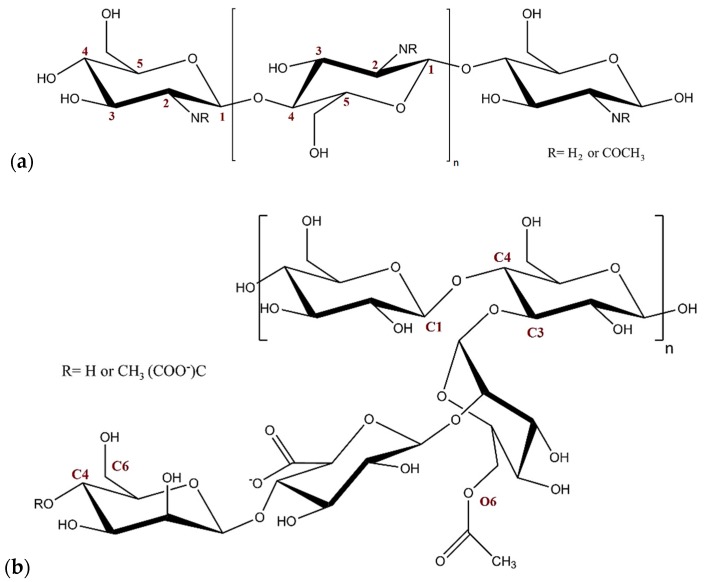
Schematic representation of the structure of: (**a**) chitosan (CS), and (**b**) xanthan gum (XG).

Chitosan (CS) is a hydrophilic, linear polysaccharide composed of repeated β-(1-4) linked units of either 2-amino-2-deoxy-β-d-glucopyranose (glucosamine) or 2-acetamido-2-deoxy-β-d-glucopyranose (glucosacetamide), depending on the degree of *N*-acetylation (DA) (Figure 1a) [4]. CS is prepared by the deacetylation of chitin, which is mainly found in crustaceans, fungi, and insects [5]. Following its preparation, various numbers of acetamide groups (C_2_H_5_NO) can be found randomly distributed at the C2 atoms along the CS chain [6]. This may result from the harsh alkaline conditions used to produce CS from chitin [7]. The physicochemical properties of CS, namely solubility and conformational flexibility, are highly affected by the DA [8]. Moreover, the distribution of the acetyl groups along the CS chain affects its behavior in solution [9]. The primary amine groups of glucosamine are responsible for the cationic nature and net positive charge of CS under acidic and neutral conditions, allowing it to interact with negatively charged poly-anionic molecules via electrostatic forces [10]. In contrast, CS is uncharged and insoluble under neutral-alkaline conditions [11]. The threshold between the soluble and insoluble states of CS lies between pH 6.2 and 6.5, which correlates with the pKa of CS in aqueous solutions [12]. It can be concluded that the solubility and complexation properties of CS are governed mainly by the degree of *N*-acetylation and pH of solution [13,14].

Xanthan gum (XG) is a natural microbial hetero-polysaccharide produced by fermentation of the micro-organism *Xanthomonas campestris* [15]. It consists of a cellulosic backbone (1-4 β-linked d-glucose units) with a side-chain consisting of two d-mannose units and one d-glucuronic acid at the C3 atom of alternating glucose units (Figure 1b) [16]. Internal d-mannose, which is connected to the backbone, has an acetyl group at O6, while approximately 50% of the terminal d-mannose forms a pyruvic acid group between position C4 and C6 [17]. Due to the presence of carboxylic groups, XG is a weak acid that behaves as an anionic polyelectrolyte at pH conditions higher than 3.1 (pKa of XG) [18]. As a consequence, it can form complexes with cationic polymers [19]. XG is a high molecular weight polysaccharide with a molecular weight range of 1 to 10 × 10^6^ Da, depending on the fermentation conditions used during production. XG is a widely used carbohydrate in the food industry, and is also beginning to be used in the pharmaceutical industry [17]. This is due to its safety in vivo, high stability over a wide range of pH and temperature conditions, and high resistance against enzymatic degradation [20].

The combination of CS and XG has been widely studied as a promising drug delivery system. Their interaction in aqueous solution results in hydrogel formation [21]. These hydrogels consist of a three-dimensional network formed via ionic interactions between the oppositely charged polymers [22]. Electrostatic attraction between the positively charged amino groups (−NH_3_^+^) of CS and the negatively charged carboxyl groups (–COO^−^) in XG is thought to be the driving force for polyelectrolyte complex formation [23,24]. The properties of the resultant complex can be altered by changing the electrostatic behavior of each polymer [25]. This can be achieved by changing either the DA of CS or the pH of aqueous solutions [26]. In addition, changing the mixing conditions (in particular the reaction time and ratio of each component) has been hypothesized to play an important role in governing the physicochemical properties and stability of the resulting complexes [26,27].

Several studies have examined the structural dynamics of chitosan utilizing molecular dynamic simulations (MDs). Prathab and Tejraj used molecular dynamics studies to determine the physical and mechanical properties of CS, chitin, and cellulose [28]. Calculations of the solubility parameter (δ) showed that CS has the highest aqueous solubility of the polymers examined. Conformational changes of CS dimers at various DA values using both molecular dynamics and Monte Carlo simulations have also been reported [29]. It was found that the most conformationally flexible disaccharide unit is the 50% acetylated form of CS, and that the intramolecular hydrogen bond between atom OH3 from one sugar unit and atom O5 from the succeeding unit is responsible for the stabilization of this conformation. Franca et al. reported that the flexibility of the polysaccharide chain is inversely related to the stability of the aforementioned intramolecular hydrogen bond [30]. Moreover, NH_3_^+^ groups seem to increase water exchange around the O3 atom, destabilizing the HO3-O5 hydrogen bond which is responsible for reducing its conformational flexibility and locking the polymer conformation into a twofold helix. In other work, Franca et al. considered the conformational flexibility and ability of CS to form nanoparticles as a function of the degree and distribution of *N*-acetyl groups [31]. It was noted that the flexibility of the CS chain is inversely proportional to its degree of acetylation. In addition, their results suggested that the block distribution of acetyl groups caused more nanoparticle aggregation than uniform distribution. The effect of water on the complexation between CS and the drug gemcitabine has been reported [32]. The loading efficiency of the drug was enhanced when water was added to the system, whereas shorter distances between CS molecules and gemcitabine were reported in the absence of water. Furthermore, MDs have been utilized to study the mechanism of nanoparticle formation between CS and tripolyphosphate (TPP) [33]. It was found that the charge properties of TPP and the coordination number of CS are the main factors in determining the energy of interaction between them.

MDs of a single chain of XG in aqueous solution showed that the side-chain direction was toward the periphery, thus shielding the XG backbone from direct contact with adjacent chains [34]. Moreover, the results revealed that the most stable conformation of XG in solution is very close to its ordered conformation in the solid state. Levy et al. examined the effect of pyruvate and acetate groups on the conformational changes of XG in aqueous solution using three XG models; wild form, acetylated, and non-acetylated modified forms [35]. GEGOP software and Metropolis Monte Carlo dynamic simulations were utilized. They concluded that the wild-type XG shows higher viscosity in solution and that its side-chains are more flexible than the acetylated and non-acetylated forms.

Although there is a substantial amount of scientific literature relating to CS and XG complexes, the intermolecular binding mechanism and conformational changes which occur at the molecular scale are still unclear. Furthermore, MDs have not, to the best of our knowledge, been utilized to elucidate the interaction between the two polymers. In addition, there are controversial findings in the literature regarding the effect of CS DA on its properties. The aim of the work reported herein is to study the behavior, from a molecular perspective, of CS in aqueous solution as a function of its protonation and the degree and position of *N*-acetylation, as well as how the foregoing factors affect the interaction of CS with XG.

## 2. Results and Discussion

### 2.1. Free CS Dimers

**Figure 2:**
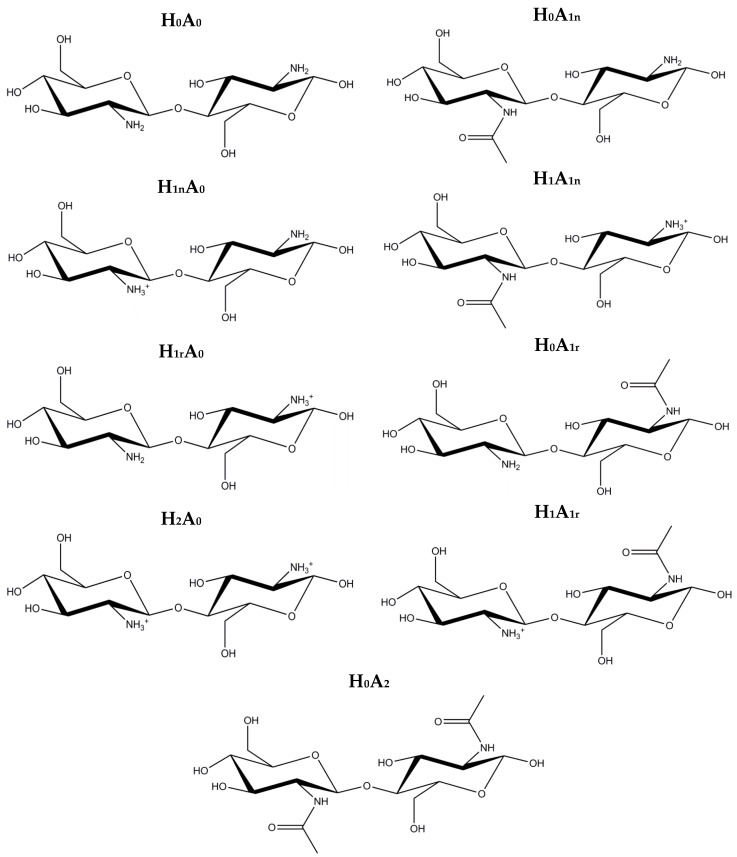
Schematic representation of chitosan (CS) dimers used in this study: (H_0_A_0_) neutral CS 0% degree of acetylation (DA); (H_1n_A_0_) CS 0% DA, 50% protonated at the non-reducing end; (H_1r_A_0_) CS 0% DA, 50% protonated at the reducing end; (H_2_A_0_) CS 0% DA, 100% protonated; (H0A1n) CS 50% DA at the non-reducing end, 0% protonated; (H_1_A_1n_) CS 50% DA at the non-reducing end, 50% protonated; (H_0_A_1r_) CS 50% DA, at the reducing end, 0% protonated; (H_1_A_1r_) CS 50% DA at the reducing end, 50% protonated; and (H_0_A_2_) CS 100% DA (chitin).

It is known that the degree of acetylation and protonation play a major role in defining the physicochemical characteristic of CS; namely its conformation, solubility, and its ability to complex with other molecular entities [23,36,37]. Therefore, nine dimers with various degrees of acetylation and protonation, either on the reducing or the non-reducing end, were considered for study (Figure 2). Subsequently, a 100 ns MD simulation was carried out for each dimer.

**Figure 3:**
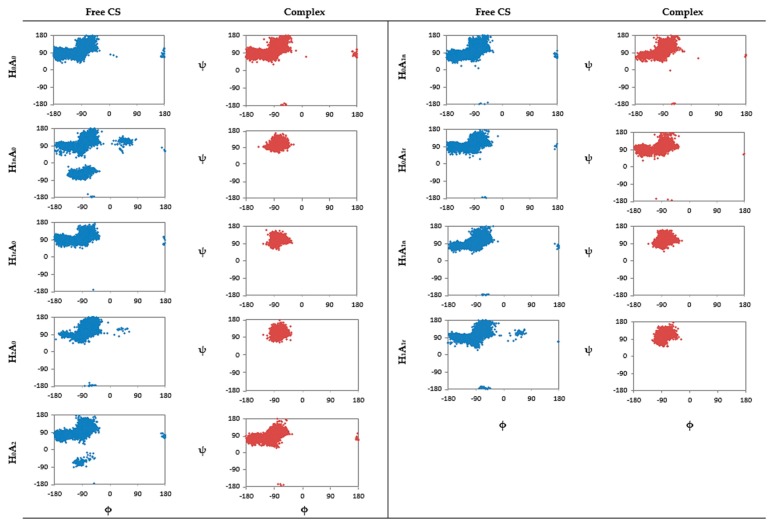
Distribution plot (x, y) for the dihedral angles (φ, **ψ**) of CS before and after complexation with XG.

**Figure 4:**
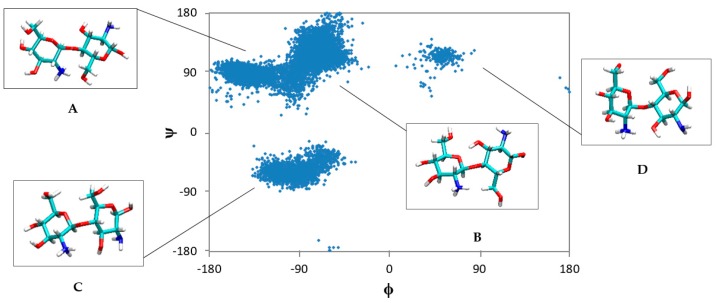
Representative snapshots from each population of H_1n_A_0_. Dihedral angles (φ, ψ) associated with each conformation are: A (−135°, 80°), B (−70°, 100°), C (−95°, −60°), and D (55°, 110°).

In order to obtain a clear image regarding the conformations that CS may adopt in aqueous solution, distribution plots of the dihedral angles, φ and ψ, of the glycosidic bond were generated from the last 60 ns of each trajectory (Figure 3). Amongst all CS dimers, it is obvious that the mono-protonated, deacetylated CS dimer (H_1n_A_0_) exhibits the broadest distribution of φ and ψ values. Four main clusters can be noticed in the plot. Representative snapshots extracted from the MD simulation for each cluster are shown in the data in Figure 4. The most populated distributions of φ and ψ (A and B) show that the protonated amine is interacting with the primary hydroxyl group on the adjacent unit, while the two-amine groups are opposite each other. For the less populated distributions (C and D), it seems that the glucose units rotate to enhance the interaction of the uncharged amine with the charged one via ion–dipole interactions. It is clear that, when the protonation is on the reducing end of the sugar (H_1r_A_0_), a narrower distribution of φ and ψ values are obtained with only two clusters adopted, which are close to the A and B conformations (Figure 4). This conformational behavior is in accord with the work of Mazeau et al., who found that the molecular flexibility of CS is more affected when the substitution at C2 is on the non-reducing end, whereas a less-pronounced effect is achieved when the substituent is located on the reducing end [38].

**Figure 5:**
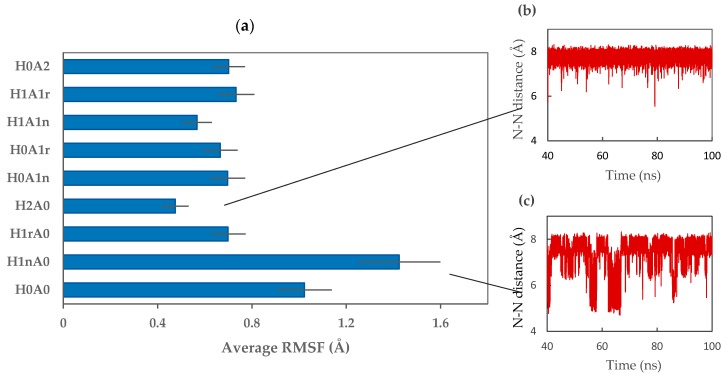
(**a**) Calculated average root mean square fluctuation (RMSF) values for the heavy atoms of each CS dimer. Error bars represent standard errors, (**b**) distance between nitrogen atoms (N-N distance) of H_2_A_0_, and (**c**) N-N distance of H_1n_A_0_.

Dihedral angle results were further confirmed by measuring the root mean square fluctuation (RMSF) of CS dimers (Figure 5a). The high average fluctuation of heavy atoms of H_1n_A_0_, which is accompanied by a high standard error value, verifies the high conformational flexibility of this dimer. Moreover, a high fluctuation in the distance between nitrogen atoms (N-N distance), starting from a distance as low as 4 Å and reaching double the distance in parts of the 100 ns simulation, can be observed (Figure 5c).

Increasing the number of protonated amines (H_2_A_0_) resulted in a decrease in conformational flexibility and a slight variation in both φ and ψ values. This was further confirmed by the low average fluctuation of H_2_A_0_ atoms, which displayed movements one third of the movement of mono-protonated H_1n_A_0_ dimers (Figure 5a). Repulsion forces between the two charged amine groups might be the reason for the reduction in both the freedom of movement and the number of possible conformations adopted by H_2_A_0_. The long distance between charged N atoms, as well as the narrow fluctuation, confirms the foregoing suggestion (Figure 5b).

On the other hand, it seems that increasing the DA to 50% or 100% does not alter the conformational distribution of the CS dimer, where two main clusters can still be detected, although a reduction in average fluctuation can be detected. The reduction is, again, more pronounced when the acetamide group is at the non-reducing end. The bulkiness of the acetamide group, together with the absence of ion–dipole interactions between protonated and deprotonated amine groups, resulted in the observed increase in rigidity compared to neutral CS (H_0_A_0_) [30,38]. However, no meaningful difference in movement restriction was detected between the half-protonated, half-acetylated, and the fully acetylated dimers, except for the H_1n_A_0_ dimer.

To further elucidate the behavior of CS in aqueous solution, and considering the major role it plays in the stabilization of the twofold helix rigid structure of chitin and β-(1-4) polysaccharides in general, intra-molecular hydrogen bonds (HB) between the O5 from one glucosamine residue with the HO3 found in the adjacent residue were studied [39]. It is thought that acetyl groups present in the structure of CS are the main contributors to the formation and stabilization of the OH3-O5 hydrogen bond [40]. In order to test this suggestion, the average distance between atoms O5 (n + 1) and H3 (n) together with the average angle formed between O3 (n)-H3 (n)-O5 (n + 1) have been calculated (Table 1).

It was found that the neutral CS dimer (H_0_A_0_) forms the weakest intramolecular hydrogen bonds with an average distance greater than 3.2 Å, an angle for O3 (n)-H3 (n)-O5 (n + 1) lower than 120°, and an occurrence, from the trajectory time, of 46%. This is in agreement with the work of Franca et al., who reported a 48% occurrence of the HO3 (n)-O5 (n + 1) HB in neutral CS [30]. It is clear that the two atoms (O3 and O5) get closer for a longer period of time when the protonation of CS is increased, forming the strongest intramolecular HB interaction with H_2_A_0_ (89%). This explains the low molecular flexibility and low RMSF values displayed by the fully protonated CS. The results obtained are in good agreement with those of Skovstrup et al., who studied the conformational flexibility of CS using MDs [29]. They found that an increasing protonation of CS resulted in a higher formation of intramolecular HBs, reaching 99% in fully protonated CS.

Increasing the degree of *N*-acetylation of CS resulted in a decrease in the distance, reaching almost 2 Å in the fully *N*-acetylated dimer (H_0_A_2_). Meanwhile, the angle for O5-H3-O3 increased, which reflects the occurrence of a more stable HB which lasts >80% of the whole trajectory time; therefore, a more rigid structure is formed. These outcomes correlate well with the RMSF results, and confirm the suggestion of a direct proportional relationship between the DA and rigidity of CS chains [40].

### 2.2. CS–XG Complexes

**Figure 6:**
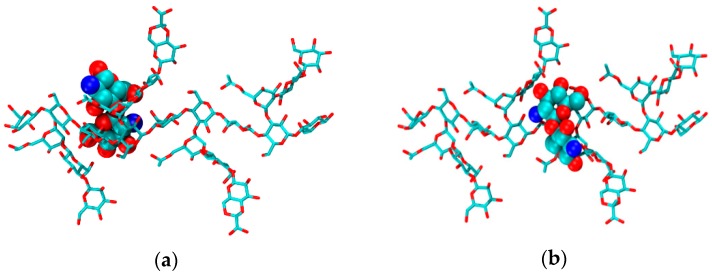
Molecular representation of the initial structure of a representative CS dimer (H_1n_A_0_) at: (**a**) the top-scored geometry produced from docking studies; and (**b**) the other possible initial structure.

The initial geometries of the 1:1 CS–XG complexes were obtained using AutoDock Vina tools [41]. The top-scored structure for each complex was considered for the subsequent MDs. Initial structures derived from docking showed that the CS is located parallel to glucose residues 3 and 4 in the backbone of XG between side-chain 1 and chain 2. The initial structure of H_1n_A_0_ is shown in (Figure 6a) as a representative dimer. It was noticed that this binding site scored top for all of the CS dimers modelled, regardless of their DA or state of protonation. In order to justify the chosen initial structures, another possibility for the H_1n_A_0_ dimer was considered in which CS is located between chains 3 and 4 of XG (Figure 6b). The results of MDs using the latter structure showed that CS moves from its initial position, reaching a position more or less close to the top-scored structure (a) after about 40 ns. Therefore, in the present study, 100 ns MDs were carried out and only the last 60 ns of each trajectory was analyzed, allowing adequate time for the CS and XG molecules to sample different geometries until reaching equilibrium.

**Figure 7:**
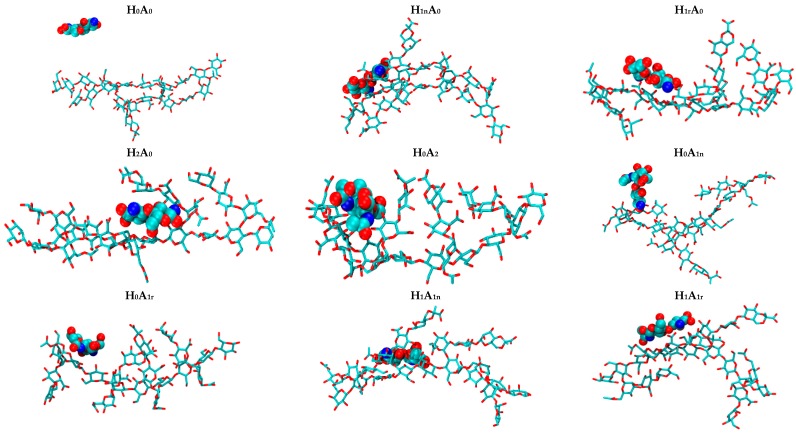
Average structure of each CS–XG complex during molecular dynamic simulations (MDs). Hydrogen atoms are excluded for clarity.

The computed average structures of each CS–XG 1:1 complex derived from the last 60 ns of the corresponding trajectories are shown in Figure 7.

**Figure 8:**
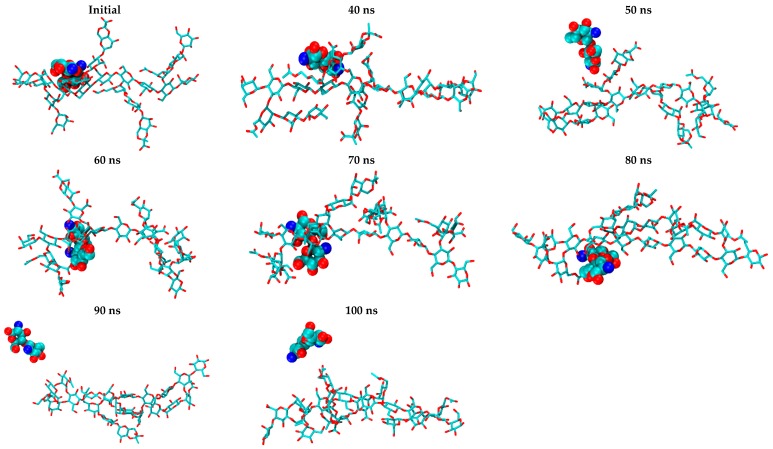
Snapshots of H_0_A_0_/XG complexes extracted from a 100 ns MD simulation.

It can be noticed from the snapshots of the uncharged CS dimer (H_0_A_0_) that it possesses a low degree of contact with XG, while more exposure to the surrounding water occurs with time. An examination of H_0_A_0_ snapshots (Figure 8) showed that CS kept moving along the XG chains during the simulation time to find the most favorable site of interaction, ending by being surrounded by more water molecules than the XG molecule. This behavior is predictable, since the amine groups present in the structure of CS are uncharged, minimizing their electrostatic interaction with the negatively charged carboxylic groups of XG.

**Figure 9:**
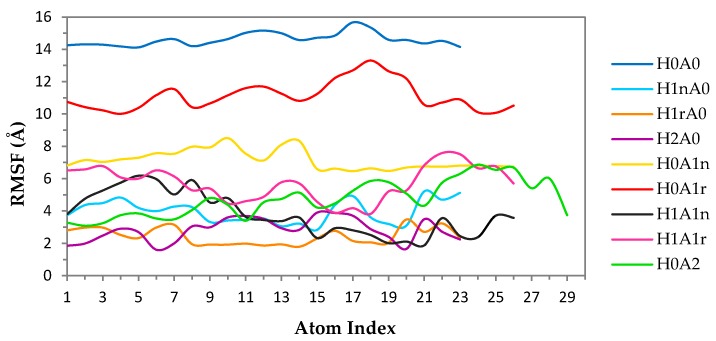
RMSF values for the heavy atoms of CS dimers obtained from the last 60 ns of the MDs of their complexes with XG.

Root mean square fluctuation (RMSF) of the heavy atoms in CS have been computed and are presented in Figure 9. The data clearly shows that the free mobility of the H_0_A_0_ dimer in the presence of XG reaches an RMSF value of about 14 Å. The average ensemble structures of the complexes of the mono-protonated dimers (H_1n_A_0_ and H_1r_A_0_) show the formation of more favorable complexes compared to H_0_A_0_ with an increase in the surface area of CS being exposed to XG (Figure 7). When the free amine group at the non-reducing end is protonated (H_1n_A_0_), CS shows a better contact with XG compared to when the reducing end is protonated (H_1r_A_0_). The structure at the end of the MD simulation time shows a noteworthy deviation of XG side-chains from their initial orientation in the H_1n_A_0_ complex, while the deviation is minor in the H_1r_A_0_ complex. An examination of the corresponding average structure of the fully protonated form (H_2_A_0_) reveals that CS favors interaction with XG. Also, it is interesting to note that H_2_A_0_ moves from its initial position after 40 ns to reach almost the middle of the XG, probably in order to interact with the anionic pyruvate groups in chains 2 and 3 in XG. The *N*-deacetylated mono- and di-protonated CS dimers show much lower RMSF values compared to the uncharged *N*-deacetylated CS (Figure 9), indicating more stable complexes.

**Figure 10:**
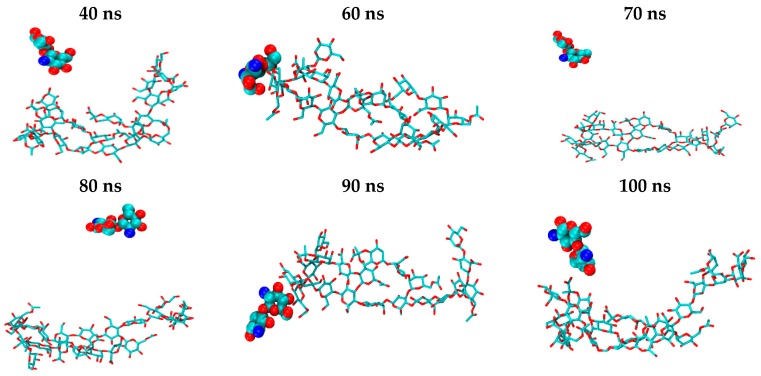
Snapshots of H_2_A_0_ complexed with neutral XG extracted from a 100 ns MD simulation.

*N*-acetylation appears to increase the mobility of uncharged CS molecules, especially in the fully *N*-acetylated dimer. This raises questions regarding the role of acetyl groups in the formation and stabilization of CS complexes. Fully *N*-acetylated CS shows a fluctuation from the initial structures in the range of 5–6 Å, which might suggest the capability of fully acetylated CS in forming stable complexes with XG more than neutral CS moieties. The behavior of complexes when XG is protonated was also considered. It is noteworthy that the interaction between CS and protonated XG is much less favored compared to the interaction with anionic XG (Figure 10).

Conformational changes of CS upon complexation with XG were evaluated and are presented in Figure 3. Interestingly, all protonated CS dimers in CS–XG complexes (whether acetylated or not) tend to be less flexible, compared to CS alone, with one population being mainly visited (the one close to A in Figure 4). The effect is much less pronounced for uncharged acetylated or deacetylated dimers of CS. It appears that the presence of a strong interaction of the protonated amine with anionic XG restricts rotation around the glycosidic linkage in the protonated dimers. Similar observations were noticed for the effect of complexation on the strength of intramolecular hydrogen bonding HO3 (n)-O5 (n + 1). Here, also, only protonated guest (CS) molecules, regardless of acetylation, show an increase in the strength of intramolecular HBs. A dramatic increase in the percentage occupancy of the intramolecular HBs was observed, reaching values >98%, while deprotonated and acetylated dimers showed a variation in HB occupancy of no more than 10%. The effect of protonation on the strength of HO3 (n)-O5 (n + 1) correlates well with the dihedral angles results (Table 1).

Since the driving force of interaction between XG and CS is thought to be electrostatic, the distances between amine groups in CS and the carboxyl groups in the adjacent side-chains of XG were calculated. Data obtained (Table 2) clearly show that the carboxylate groups on chain 2 of XG are generally closer to the protonated amine groups than to the free amine, with a lower standard deviation. Free amine groups in the uncharged dimer H_0_A_0_ are located a far distance from both carboxylic groups, with high fluctuation and standard deviation, reaching up to 9 Å. However, introducing acetamide groups considerably lowers the standard deviation values. *N*-acetylated CS dimers showed distance values in between protonated and deprotonated dimers, with a maximum deviation of 5 Å, which may indicate the occurrence of van der Waals interactions between acetamide groups and carboxylate groups.

The data for the intermolecular hydrogen bonds (HBs), which exist between each CS dimer, are presented in Table 3. Likewise, guest-host (CS–XG) intermolecular hydrogen bonds were monitored. Free guest dimer molecules form several HBs with the surrounding water molecules. A strong reduction was observed in the number of water-guest HBs for chitosan upon complexation, resulting in a less flexible and more packed conformation (Figure 3). The reduction was more evident in protonated CS molecules. This indicates that protonated CS dimers, regardless of their DA, are less exposed to the surrounding water. It seems that CS molecules are forming HBs with XG. Hence, they are more buried in the XG bulk structure. *N*-deacetylated, uncharged CS dimers form the minimum number of intermolecular HBs with XG (Table 3).

### 2.3. MM-PBSA

The data in Table 4 lists the binding free energy (∆G) values together with other energy terms that contribute to the free energy of the studied complexes computed using MM-PBSA. It is noticeable that electrostatic interactions are vital contributors to the stability of CS–XG complexes. This becomes more recognizable when raising the number of protonated amines in CS, being most favorable in the fully protonated complex (H_2_A_0_/XG, ∆E_ele_ = −419 kcal/mol). This is a reasonable assertion, since introducing ammonium groups will increase the positive charge of CS. Accordingly, electrostatic interactions between CS and XG will increase owing to the negatively charged carboxyl groups present in XG. The data in Table 4 demonstrate that van der Waals forces contribute to the stability of the CS–XG complex; van der Waals forces are least favorable under neutral conditions (H_0_A_0_), attaining a value of ∆E_vdW_ = −14.03 kcal/mol.

Non-polar forces, ΔG_NP_, are also favorable in all CS–XG complexes, showing values around −3 kcal/mol, indicating a positive contribution for the non-polar surface-accessible area on the stability of the complexes formed, though to a much lower extent compared to the electrostatic and van der Waals interactions. MM-PBSA calculations also indicate a reduction in electrostatic interactions between both CS and XG with water molecules as a result of complexation, leading to a large, unfavorable electrostatic solvation energy (ΔG_PB_ is positive). This is more pronounced in the case of protonated dimers, reaching a maximum of 416 kcal/mol in H_2_A_0_, due to the fact that the desolvation of the charged species requires more energy than the neutral one. Similarly, it is noticeable that when amine groups are uncharged, ΔG_PB_ becomes less positive. Overall, the total solvation free energy (∆G_solv_) for both polar and non-polar areas is positive for all complexes demonstrating the formation of insoluble polyelectrolyte complexes between CS and XG; the insolubility of the resulting complexes increased with protonation. Data acquired from the MD simulations show that the protonated amine at the reduced position still partially interacted with the surrounding water molecules.

Normal mode analysis shows negative values of the configuration entropy (∆S_conf_) for all complexes examined, thus indicating a reduction in the conformational flexibility of both the guest and host and freedom upon complexation. The trend for ∆S_conf_ values are in accord with the binding free energy values (enthalpy-entropy compensation).

*N*-acetylated, non-protonated dimers form more stable complexes with XG than H_0_A_0_ by approximately 4 kcal/mol. It is possible that the acetamide group is then able to penetrate between the XG chains, leading to further interactions between CS and XG. The role of the acetamide group is verified by the increase in contribution from van der Waals forces with an increasing number of acetyl groups. Fully *N*-acetylated CS dimers formed the most favorable van der Waals interaction with XG, ∆E_vdW_ = −25 kcal/mol. The total number of solute–substrate interactions (ΔE) is higher when the acetyl group is introduced in the non-reduced position (H_0_A_1n_) compared to the reduced position (H_0_A_1r_). Again, the effect of placing the acetamide or amine groups in the reduced or non-reduced position affects the strength of intermolecular interactions as well as the geometries of the CS–XG complex. The foregoing observation will be the subject of a future study, but modelled using longer CS chains with more diverse possibilities in relation to the distribution of *N*-acetyl groups.

The results clearly show, as expected, that the value of ΔG becomes more negative as the number of protonated groups increases (H_0_A_0_, H_1_A_0_, and H_2_A_0_ complexes), due to the interaction of the protonated amine with the anionic chains of XG. However, the difference in ΔG value between H_2_A_0_ and H_1_A_0_ complexes is less than 2 kcal/mol, despite the increase in the value of ΔE_ele_ when moving from the mono- to the di-protonated species. This increase in ΔE_ele_ is offset by the extent of the unfavorable desolvation for the H_2_A_0_ complex compared to the H_1_A_0_ complex. The position of the ammonium group has an impact on the strength of the electrostatic interactions (H_1n_A_0_ and H_1r_A_0_). This is illustrated by the fact that the ΔE_ele_ value in half-protonated CS is more favorable when protonation is at the non-reduced end of the glucosamine unit of CS, because the electrostatic force is the most dominant force in this interaction. Again, the desolvation process counterbalances this effect, and the difference in ∆G between H_1n_A_0_ and H_1r_A_0_ is ~1 kcal/mol.

The influence of the molecular weight of XG on the extent of the CS–XG interactions has been addressed. MDs of H_1n_A_0_ with XG composed of two monomers (H_1n_A_0_/2XG) were performed. A remarkable reduction in the binding free energy, ΔG, of around 7 kcal/mol compared to the four-monomer XG complex with H_1n_A_0_ was observed. This finding may suggest a positive contribution of other XG chains (chains 3 and 4) on complex stability.

## 3. Computational Methods

The initial structure of XG was obtained from PolySac3DB, a database of three-dimensional (3D) polysaccharides structures, which was deduced from X-ray diffraction results and computer-aided model building [42,43], whereas the CS structure was generated using HyperChem™ Professional 7.51 [44]. A total of nine CS dimers were built to cover a wide range of *N*-acetylation and protonation states (Figure 2). Free amine groups present in the CS backbone were substituted by acetamide groups (C_2_H_5_NO) in order to increase the degree of *N*-acetylation, whereas hydrogen atoms were added to the existing free amine groups (−NH_3_^+^) to form protonated CS molecules [45]. A ligand flexible docking study was performed to find the possible binding sites and binding affinities of CS using AutoDock Vina [41], where XG was assigned as the host molecule and CS as the guest. The simulation box used was sufficiently large to include both CS and XG.

MDs were performed using AMBER 11 software [46] employing the Parm99 force field, which includes a developed parameter set for polysaccharides [47,48]. The atomic charges for the host and guest molecules were obtained using AM1-BCC charge sets [49]. Each system, for both the free CS species and their corresponding CS–XG complexes, was solvated in a periodic box of a TIP3P water model [50]. Chloride and sodium ions were added, when needed, in order to maintain the neutrality of the systems [51]. Periodic boundary conditions were adopted, and the Particle Mesh Ewald (PME) method was used for the treatment of long-range electrostatic interactions [52]. The non-bonded cutoff was set to 10.0 Å. Before starting the MDs simulations, each system was subjected to energy minimization, then heated up to 298 K for 60 ps. A total of 100 ns MD simulation runs were carried out. The system was coupled in the NPT ensemble to a Berendsen thermostat at 298 K and a barostat at 1 atm. A 2-fs time step was used with structures being saved every 2 ps.

MDs analyses were performed using the PTRAJ module of the AMBER 11 tools [46]. Visualization of the outputs and MD calculations were undertaken using VMD 1.8.6 software [53]. In order to obtain physically meaning average structures, the kclust tool was used from the MMTSB package [54]. A large cut-off was set to include all snapshots extracted from the corresponding trajectories in one cluster, and the structure that was nearest to the cluster center was considered the average structure.

**Figure 11:**
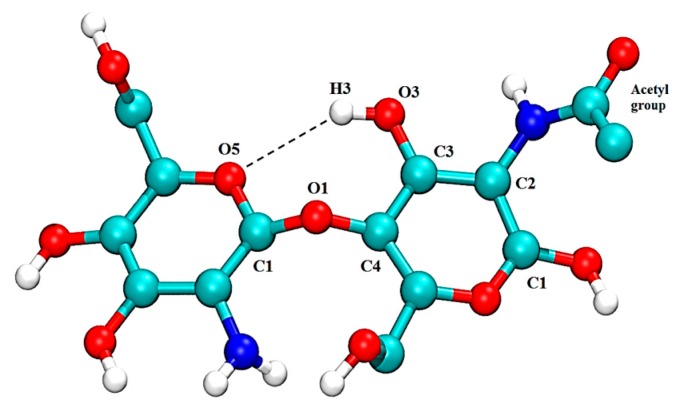
Molecular representation of a CS dimer, 50% acetylated, with φ and ψ dihedral angles defined by atoms O5-C1-O1-C4 and C1-O1-C4-C3, respectively. The dashed line represents the intramolecular hydrogen bond OH3-O5.

Conformational changes within the structure of CS before and after complexation were studied by determining the torsional (dihedral) angles around the glycosidic bond. Phi (φ) and psi (ψ) dihedral angles were defined by the planes formed via atoms O_5_-C_1_-O_1_-C_4_ and C_1_-O_1_-C_4_-C_3_, respectively, as illustrated in Figure 11 [31].

For hydrogen bond evaluation, a hydrogen bond was defined as a donor–acceptor interaction where the distance between the acceptor atom and the donor hydrogen atom is below 3 Å. The angle between donor and acceptor was set to be greater than 120° [55].

The molecular mechanics/Poisson–Boltzmann surface area (MM-PBSA) was utilized to calculate the binding free energy (∆G) for each complex as implemented in AMBER 11 tools [46]. Analytical methods and equations utilized have been described in detail elsewhere [56,57]. A short description of the equations is given here. The free energy of binding (∆G) was estimated according to the following expression:

∆G = ∆E_gas_ + ∆G_solv_ (1)

where ∆E_gas_ is the gas phase interaction energy between the guest and host, and is approximated by the sum of the following energy changes upon complexation: the internal energy (∆E_INT_), the van der Waals interaction energy (∆E_vdW_), and the electrostatic interaction energy (∆E_elec_), as follows:

∆E_gas_ = ∆E_elec_ + ∆E_vdW_ + ∆E_INT_. (2)

The solvation free energy, ∆G_solv_, can be subdivided into electrostatic or polar (∆G_PB_) and non-polar (∆G_NP_) components, as follows:

∆G_solv_ = ∆G_PB_ + ∆G_NP_. (3)

The free energy, ∆G_PB_, was computed in a continuum solvent using the MM-PBSA program in AMBER 11, whereas ∆G_NP_ was calculated from the solvent-accessible surface area (SASA) using the following terms:

∆G_NP_ = γ SASA + b (4)

where γ = 0.00542 kcal/(mol Å^2^), and b = 0.92 kcal/ mol.

## 4. Conclusions

The behavior of solvated CS together with its complexation with XG was addressed by means of MDs and MM-PBSA studies. Three levels of acetylation and protonation states were chosen (0, 50%, and 100%) for CS. Complexes of CS–XG indicate the preference of CS to interact and penetrate in between the chains of XG when protonation is increased. The average structures of CS–XG complexes show close contact and more favorable interaction between the two polymers when CS is 50–100% protonated, whilst deprotonated CS interacts preferentially with bulk water molecules more than XG.

MM-PBSA calculations revealed that electrostatic forces (polar interactions) were of major importance for the stability of the formed CS–XG complexes, and that the strength of this interaction is related to the protonation state of CS. The results also showed that not only does the protonation state affect the binding free energy of complexes, but the position of the ammonium groups plays an important role in the interaction between CS and XG as well. In addition to protonation, acetamide groups in the structure of CS play a role in the formation and stabilization of its corresponding complexes with XG.

It can be concluded that the pH of the aqueous solution (protonation state of CS) is the predominant factor in determining the extent of interaction between XG and CS, and that the position of ammonium and acetyl groups plays a major role in the stabilization of the complexes formed.

## Figures and Tables

**Table 1:** Average HO3 (n)-O5 (n + 1) distances and O3 (n)-H3 (n)-O5 (n + 1) angles for CS dimers before and after complexation with XG ^a^.

	Free CS			CS–XG Complex		
	Distance (Å)	Angle (°)	%Occupancy	Distance (Å)	Angle (°)	%Occupancy
**H_0_A_0_**	3.25 (1.19)	94.601 (54.9)	46.72	3.10 (1.17)	99.703 (54.6)	53.37
**H_1n_A_0_**	3.05 (1.30)	127.16 (51.6)	60.58	1.90 (0.32)	152.26 (19.9)	98.67
**H_1r_A_0_**	2.47 (0.90)	132.74 (35.2)	72.87	1.85 (0.19)	157.57 (11.8)	99.86
**H_2_A_0_**	2.62 (0.56)	134.09 (26.8)	89.21	1.95 (0.28)	151.32 (18.1)	99.32
**H_0_A_1n_**	2.38 (0.93)	129.65 (45.9)	80.84	2.17 (0.73)	139.34 (40.3)	88.76
**H_0_A_1r_**	2.33 (0.88)	137.83 (38.3)	80.94	2.52 (1.02)	131.80 (42.6)	78.33
**H_1_A_1n_**	2.13 (0.52)	145.88 (24.2)	84.76	1.88 (0.28)	152.56 (16.9)	99.63
**H_1_A_1r_**	2.29 (0.93)	131.09 (43.0)	82.40	1.93 (0.29)	150.74 (19.6)	98.66
**H_0_A_2_**	2.12 (0.76)	144.68 (33.9)	89.61	2.20 (0.79)	140.78 (36.5)	85.39

^a^ Numbers in parentheses represent standard deviations of the results.

**Table 2:** Average distances (Å) between carboxylic groups in chain 2 of XG, and nitrogen atoms in CS for each complex.

	Pyruvate COO^−^		Glucoronate COO^−^	
	N1	N2	N1	N2
**H_0_A_0_**	22.40 ± 9.09	21.26 ± 8.74	18.17 ± 9.25	17.63 ± 8.62
**H_1n_A_0_**	13.29 ± 0.96	18.99 ± 1.96	7.151 ± 0.74	13.88 ± 0.99
**H_1r_A_0_**	12.91 ± 0.93	19.64 ± 1.48	6.550 ± 1.23	13.73 ± 1.27
**H_2_A_0_**	11.50 ± 2.57	9.350 ± 3.45	6.942 ± 1.89	10.66 ± 2.31
**H_0_A_1n_**	27.21 ± 3.49	22.87 ± 2.75	19.67 ± 4.01	16.65 ± 2.91
**H_0_A_1r_**	17.95 ± 4.21	16.02 ± 4.12	16.88 ± 5.29	14.06 ± 3.81
**H_1_A_1n_**	15.87 ± 2.99	13.86 ± 1.18	12.90 ± 1.98	7.002 ± 1.12
**H_1_A_1r_**	15.37 ± 0.67	9.710 ± 1.01	7.998 ± 1.16	4.021 ± 0.75
**H_0_A_2_**	12.57 ± 5.14	14.80 ± 3.41	11.33 ± 3.48	9.052 ± 3.92

**Table 3:** Water-guest (CS) and host (XG)-guest (CS) intermolecular hydrogen bond analysis ^a^ for free CS and CS–XG complexes.

	Free CS	Complex	
	CS-H_2_O	CS-H_2_O	CS–XG ^b^
**H_0_A_0_**	16.9	8.4	2.4
**H_1n_A_0_**	16.3	4.7	4.9
**H_1r_A_0_**	15.0	5.0	4.3
**H_2_A_0_**	15.8	2.0	4.5
**H_0_A_1n_**	16.9	9.5	3.7
**H_0_A_1r_**	16.9	8.5	2.4
**H_1_A_1n_**	16.5	5.4	4.3
**H_1_A_1r_**	16.3	5.4	4.4
**H_0_A_2_**	16.9	8.8	3.8

^a^ The numbers in the Table represent the average number of hydrogen bonds formed during the specified MMS time. ^b^ Number of hydrogen bonds between CS and XG in water.

**Table 4:** MM-PBSA results for the nine CS dimer complexes with the four-monomer XG molecule examined (the values presented are in kcal/mol).

	∆E_ele_	∆E_vdW_	∆G_NP_	∆G_PB_	∆G_sol_	∆E	T∆S_conf_	∆G
**H_0_A_0_**	−21.29	−14.03	−2.59	24.47	21.89	−13.43	−12.52	−0.91
**H_1n_A_0_**	−227.53	−24.47	−3.71	226.49	222.77	−29.22	−18.36	−10.86
**H_1r_A_0_**	−208.76	−21.61	−3.45	205.63	202.18	−28.19	−17.45	−10.74
**H_2_A_0_**	−419.95	−23.27	−3.89	416.46	412.57	−30.65	−18.25	−12.4
**H_0_A_1n_**	−41.82	−17.36	−3.07	41.09	38.02	−21.16	−15.93	−5.23
**H_0_A_1r_**	−25.08	−21.12	−3.19	31.65	28.46	−17.74	−13.24	−4.50
**H_1r_A_1n_**	−192.15	−23.13	−3.53	195.25	191.72	−23.56	−15.58	−7.98
**H_1n_A_1r_**	−232.68	−23.96	−3.53	230.89	227.36	−29.28	−18.03	−11.25
**H_0_A_2_**	−25.07	−25.79	−3.63	33.78	30.15	−20.71	−16.00	−4.71
H_1n_A_0_-XG2 *	−164.11	−11.16	−2.76	159.25	156.49	−18.78	−14.83	−3.95

* This complex represents the interaction between H_1n_A_0_ dimer and a two-monomer XG molecule.

## Abbreviations

CS, chitosan; XG, xanthan gum; MDs, molecular dynamics simulations; DA, degree of *N*-acetylation; MM-PBSA, molecular mechanics Poisson–Boltzmann surface area; HB(s), hydrogen bond(s); (H_0_A_0_) neutral CS 0% DA; (H_1n_A_0_) CS 0% DA, 50% protonated at the non-reducing end; (H_1r_A_0_) CS 0% DA, 50% protonated at the reducing end; (H_2_A_0_) CS 0% DA, 100% protonated; (H_0_A_1n_) CS 50% DA at the non-reducing end, 0% protonated; (H_1_A_1n_) CS 50% DA at the non-reducing end, 50% protonated; (H_0_A_1r_) CS 50% DA, at the reducing end, 0% protonated; (H_1_A_1r_) CS 50% DA at the reducing end, 50% protonated and (H_0_A_2_) CS 100% DA (chitin).
